# Induction of Apoptotic Cell Death in Non-Small-Cell Lung Cancer Cells by MP28 Peptide Derived from *Bryopsis plumosa*

**DOI:** 10.3390/md23120481

**Published:** 2025-12-17

**Authors:** Heabin Kim, Seung-Hyun Jung, Seonmi Jo, Jong Won Han, Jei Ha Lee

**Affiliations:** 1 Department of Bio-Material Research, National Marine Biodiversity Institute of Korea, Seocheon 33662, Republic of Korea; khb7116@mabik.re.kr (H.K.); zebrajung@mabik.re.kr (S.-H.J.); 2 Department of Biological Application and Technology, National Marine Biodiversity Institute of Korea, Seocheon 33662, Republic of Korea; joseonmi@mabik.re.kr; 3 Department of Ecology & Conservation, National Marine Biodiversity Institute of Korea, Seocheon 33662, Republic of Korea; jwhan@mabik.re.kr

**Keywords:** anticancer peptide, *Bryopsis plumosa*, non-small-cell lung cancer, apoptosis, EMT

## Abstract

Marine algae are a prolific bioactive peptide source with a broad pharmacological potential. We characterized MP28, a cationic peptide isolated from the green alga *Bryopsis plumosa*. Structural modeling indicated a predominantly amphipathic α-helix (residues 3–16) flanked by flexible termini and stabilized by intramolecular disulfide bonds, a motif typical of membrane-active anticancer peptides. Functionally, MP28 demonstrated potent activity against non-small-cell lung cancer cell lines (A549, H460, H1299) without affecting non-tumorigenic lung fibroblasts (MRC-5). In vitro, MP28 decreased cell viability and clonogenic growth and suppressed migration and invasion in a dose-dependent manner. Flow cytometry revealed increased early/late apoptotic fractions, accompanied by caspase-9 activation, consistent with engagement of the intrinsic apoptotic pathway. In a mouse xenograft model, MP28 treatment significantly reduced tumor size compared with that of controls. Collectively, MP28 may be a potent anticancer peptide that exhibits selective cytotoxicity and low toxicity toward normal cells.

## 1. Introduction

Lung cancer is a prevalent disease that imposes a massive burden on the world’s population because of its high mortality rate [1]. In 2022, lung cancer accounted for approximately 2.48 million new cases and 1.8 million deaths worldwide. Assuming that incidence and mortality rates remain unchanged from 2022, the number of lung cancer cases and deaths is anticipated to increase to 4.62 million and 3.55 million by 2050. [2]. Non-small-cell lung cancer (NSCLC) accounts for approximately 80% of all lung cancer cases, with lung adenocarcinoma and lung squamous cell carcinoma as the predominant histological subtypes [3]. Incidence and mortality are significantly higher in men than in women with lung cancer, reflecting sex-related disparities associated with differences in smoking behavior, genetic susceptibility, and potential hormonal influences [4,5]. Like other malignancies, lung cancer acquires the ability to evade apoptosis during its progression toward cellular immortality, which is closely linked to therapeutic responsiveness [6]. Although conventional chemotherapeutic agents focus on inducing apoptosis, they face persistent drug resistance and adverse effects [7,8]. Multi treatment strategies, including chemo/radiotherapy, surgery, targeted therapy, and immunotherapy have improved clinical outcomes; however, they are often accompanied by considerable toxicity and high costs, and the emergence of drug resistance remains a major barrier in clinical management [9]. These limitations have accelerated the search for safer and novel therapeutics to alleviate the clinical burden of lung cancer.

Anticancer peptides (ACPs) are powerful candidate agents capable of eliminating cancer cells by disrupting mitochondria or through membranolytic mechanisms [10,11]. Owing to the net negative charge of cancer cell membranes compared to that of normal eukaryotic membranes, peptides have emerged as next-generation anticancer candidates characterized by high selectivity, low systemic toxicity, and structural diversity [12]. Specifically, they can penetrate malignant cell membranes, guided by their amphiphilicity and extent of their hydrophobic arcs, and compromise membrane integrity. 

Evidence from multiple tumors demonstrates that anticancer peptides (ACPs) frequently trigger apoptosis [13,14,15]. In lung cancer, the designed peptide, AC-P19M, induced apoptosis and suppressed cell migration and invasion [16]. Cationic AMP D-LAK-120A reduces NSCLC cell proliferation and migration while increasing apoptosis in vitro [17]. A peptide–drug conjugate, DTX-P7 (docetaxel linked to the Hsp90-targeting heptapeptide P7), enhances intratumoral action by eliciting an unfolded protein response and promoting cooperative, effective apoptosis [18]. Collectively, these findings suggest that ACPs are promising agents for reactivating cell death programs and countering metastatic traits in lung cancer.

*Bryopsis plumosa*, a common marine green alga, produces several active components with various functional structures and biological properties, including polyphenols, peptides, and polysaccharides. These biological components exhibit potential for interesting textural, gelling, antioxidant, antimicrobial, and anticancer properties. We previously found that the *Bryopsis plumosa*-derived peptide MP06 exerts minimal effects on normal human lung fibroblasts while suppressing the growth, invasion, and metastasis of lung cancer cells [19]; moreover, it inhibited angiogenesis, as validated in human umbilical vein endothelial cell assays [20]. Therefore, peptides are tumor-selective agents that induce apoptosis in cancer cells.

Here, we report the discovery of an MP28 peptide with strong and selective anticancer activity against NSCLC cells. This study aimed to evaluate the anticancer potential of MP28, including its apoptosis-inducing activity, and to validate its ability to inhibit tumor growth in vitro and in a mouse xenograft model.

## 2. Results

### 2.1. Characteristics of MP28

**Figure 1:**
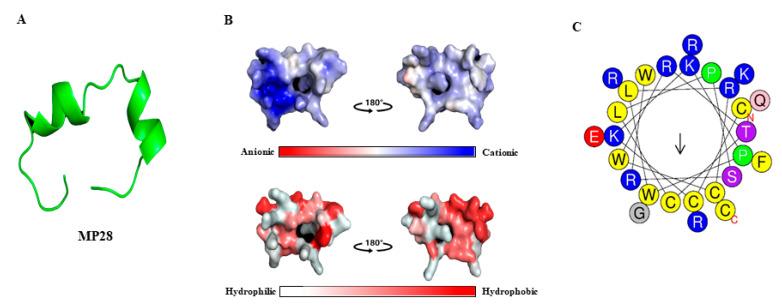
Three-dimensional model, physical properties, and helical wheel diagram of anticancer peptide MP28. (**A**) Three-dimensional model of MP28 using PEP-FOLD3 server. (**B**) Electrostatic potential and hydrophobic/hydrophilic regions of MP28. MP28 has visualized blue regions (cationic) and amphipathic (red and white) properties of anticancer peptides. (**C**) The helical wheel project for MP28 was depicted using a web tool (https://heliquest.ipmc.cnrs.fr/, accessed on 24 March 2024). Amino acids in blue color are positively charged, and those in yellow color are hydrophobic.

The three-dimensional model of MP28 (CCKKPWLRCWRTCLPSRWQRERFGRKC-NH2) was predicted using PEP-FOLD3 server [21]. MP28 consists of two α-helices, an unstructured middle, and N- and C-terminal regions, with several basic amino acids that confer a net positive charge (Figure 1A). Negatively, positively, and neutrally charged surfaces are shown in red, blue, and white, respectively. The amphipathic character is evident from hydrophobic and hydrophilic regions distributed along the peptide chain. MP28 exhibits cationic and amphipathic properties characteristic of anticancer peptides, as visualized in Figure 1B. The MP28 peptide displays moderate hydrophobicity (H = 0.423) and a relatively low hydrophobic moment (µH = 0.289), indicating a partially amphipathic α-helical character. Among the 27 amino acid residues, 14 (51.85%) are polar, including one each of Gln, Ser, Thr, and Gly, and 13 (48.15%) are nonpolar, as calculated using the HeliQuest freeware [22] (Figure 1C). The sequence contains multiple aromatic residues (three Trp and one Phe) that may contribute to membrane interactions or hydrophobic stacking. The net positive charge is +8, arising from six Arg and three Lys residues, counterbalanced by one Glu residue. Overall, this composition indicates a cationic, amphipathic peptide with a physicochemical profile commonly associated with membrane-active or anticancer peptides. 

### 2.2. Cellular Viability in Lung Cancer Cells 

**Figure 2:**
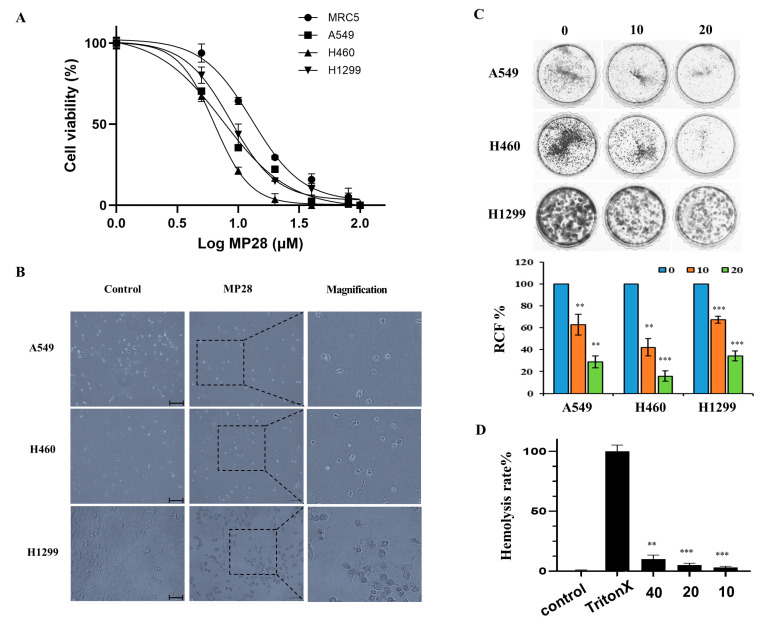
Cellular viability of MP28 in lung cancer cells. (**A**) Quantification of CCK-8 assay for non-cancerous cells (MRC5) and lung cancer cells (A549, H460, and H1299) treated with a variety of MP28 (1–100 μM). (**B**) Morphologic changes in lung cancer cells treated with 10 μM MP28 induced lung cancer cell apoptosis. Scale bar: 200 µm. (**C**) The colony formation assay was performed to determine the association of MP28 with tumor growth. (**D**) Hemolysis ability analyzed using red blood cells treated with MP28 (10–40 µM) for 35 min at 37 °C. Cells treated with 1% Triton X-100 were used as positive controls and set as 100% hemolysis. ** *p* < 0.01; *** *p* < 0.001.

To study the effect of MP28 on NSCLC, we evaluated its cytotoxicity on MRC-5 lung fibroblasts and cancer cell lines (A549, H460, and H1299) using the CCK-8 assay. Treatment with increasing MP28 concentrations significantly reduced cell viability of lung cancer cells compared with that of non-cancerous MRC-5 cells (Figure 2A). The IC_50_ values of MP28 were MRC-5 (13 ± 1.6 μM), A549 (7.5 ± 0.8 μM), H460 (6.3 ± 0.2 μM) and H1299 (8.6 ± 0.8 μM), indicating higher sensitivity of cancer cells to peptide. Additionally, lung cancer cells treated with 10 μM MP28 demonstrated morphological changes from a spindle to a cobblestone-like shape, indicative of typical cell death (Figure 2B). Accordingly, MP28 was more cytotoxic to cancer cells than fibroblasts, reflecting its selective anticancer activity. Consistent with these findings, colony formation assay showed that at 10 and 20 μM, markedly reduced cell growth in all NSCLCs(Figure 2C), indicating a significant inhibitory effect on long term cell proliferation. To assess the safety of MP28, we measured its hemolytic activity. As displayed in Figure 2D, the hemolytic activity values were 9.7%, 5.3%, and 3.1% for MP28 at 40, 20, and 10 μM, respectively. At all tested concentrations, hemolysis remained below 10% Triton X-treated positive control. Notably, MP28 caused ≤5% hemolysis at 10–20 μM, a range that is generally considered safe and unlikely to interfere with in cellular proliferation.

### 2.3. Regulation of Epithelial–Mesenchymal Transition (EMT) on MP28

**Figure 3:**
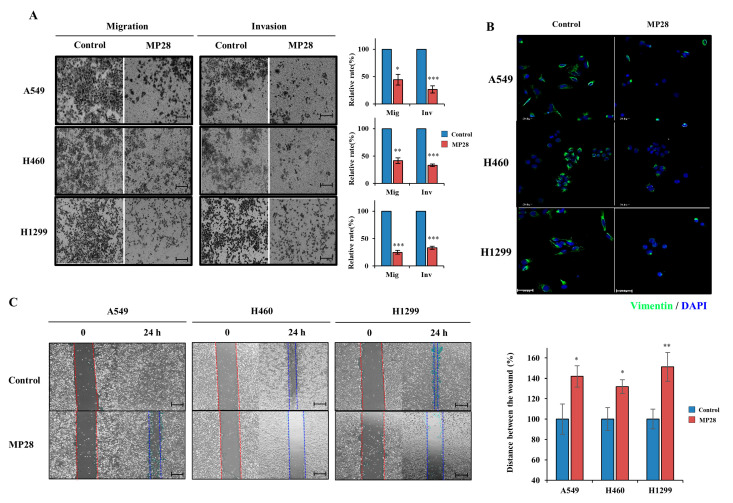
Regulation of epithelial–mesenchymal transition (EMT) on MP28. (**A**) The migration/invasion assay was performed through the transwell chambers, and peptide-treated cells exhibited a significant decrease in migration and invasion abilities. Scale bar: 200 µm. (**B**) Suppressed cellular Vimentin (green) levels with 10 μM MP28 by immunofluorescence staining. Scale bar: 50 µm. (**C**) The effect of MP28 on lung cancer cells was assessed using a wound-healing assay at 24 h. Scale bar: 200 µm. * *p* < 0.05; ** *p* < 0.01; *** *p* < 0.001.

To determine whether MP28 affects EMT in lung cancer, we performed migration and invasion assays in NSCLC cells. MP28 significantly reduced both the migratory and invasive capacities (Figure 3A). Furthermore, MP28 decreased the levels of cellular vimentin, a mesenchymal marker associated with EMT-driven motility and aggressiveness (Figure 3B). Consistently, wound-healing assays revealed larger residual wound areas in the MP28-treated groups than in the controls, indicating impaired collective migration with a more pronounced inhibitory effect at 24 h (Figure 3C). Collectively, treatment with 10 μM markedly attenuated EMT-associated migratory and invasive properties in NSCLCs.

### 2.4. Induction of Cell Apoptosis on MP28

**Figure 4:**
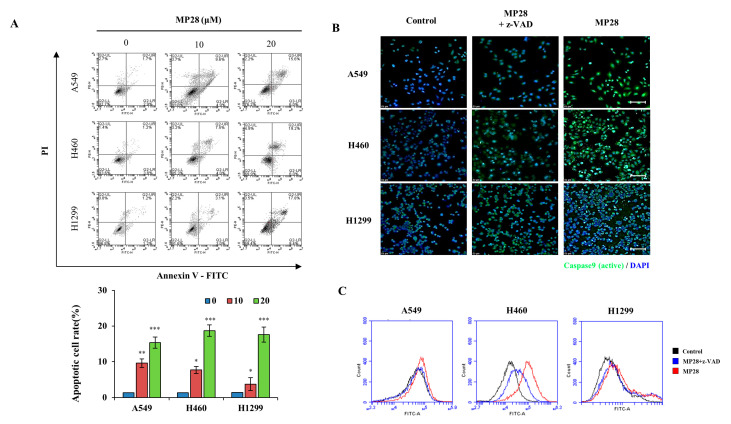
The effect of MP28 on apoptosis and caspase activity in lung cancer cells. (**A**) To evaluate whether treatment with MP28 induced apoptosis, an Annexin V/PI staining assay was performed. (**B**) Caspase-9 activation of lung cancer cells by MP28 occurs across a broad range of cells and is important for proper signaling in the apoptotic response. Here, z-VAD is recruited to caspase-9 expression, resulting in decreased expression in cancer cells with MP28. Scale bar: 100 µm. (**C**) Stained cells were analyzed for quantification by flow cytometry using the FL-1 channel. * *p* < 0.05; ** *p* < 0.01; *** *p* < 0.001.

To assess the impact of MP28 on cell apoptosis, we performed Annexin V/PI and active caspase-9 (FITC) staining assays in NSCLCs. As shown in Figure 4A, treatment with 10 and 20 μM MP28 increased apoptotic cell populations in a dose-dependent manner compared with the control group. As the concentration of MP28 increased, the percentage of apoptotic cells increased from 9.6% to 15.6% in A549 cells, from 7.5% to 19.2% in H460 cells, and from 3.1% to 17.8% in H1299 cells, with a significant increase in late apoptotic fraction. Additionally, treatment with 10 μM MP28 for 3 h triggered caspase-9 activation. Under a fluorescence microscope using an FITC filter, MP28-treated cells exhibited stronger green fluorescence signals, whereas the control and z-VAD (caspase inhibitor) groups showed weaker signals. To further substantiate these findings, fluorescence intensity was quantified using the FITC channel on a flow cytometer. The MP28-treated groups (red) displayed a rightward shift in fluorescence compared with the control (black) and the inhibitor treated groups (blue). Therefore, MP28 induces cancer cell apoptosis through the caspase-9 signaling pathway.

### 2.5. In Vivo Antitumor Effect and Safety of MP28

**Figure 5:**
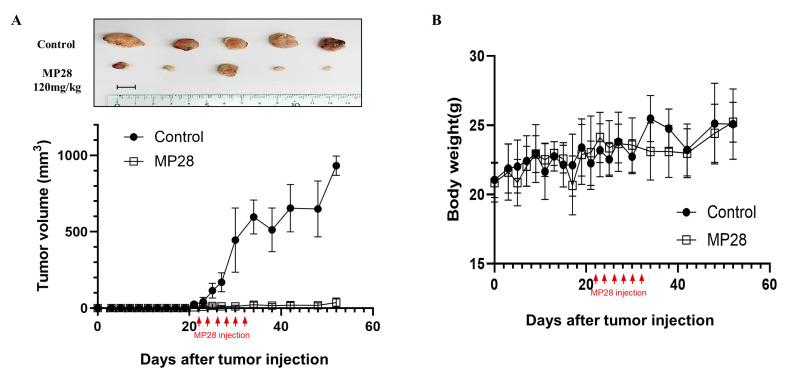
Inhibition of tumor growth with MP28 in the xenograft model. (**A**) Tumor volumes for each group calculated from regular measurements of the tumor. After tumor formation in Balb/c nude mice, MP28 was injected intratumorally 6 times at 2-day intervals (each injection dose was 20 mg/kg). Tumor burdens were removed from mice after 8 weeks of cell injections. Scale bar: 10 mm. (**B**) Changes in body weight of mice in each group during the treatment cycle.

To assess the in vivo anticancer effects of MP28, we subcutaneously implanted A549 cells into BALB/c nude mice. MP28(20 mg/kg per dose) was administered intratumorally every other day for a total of six injections. Tumors were excised 8 weeks after cell implantation. Although tumor volumes increased over time in all mice, tumor growth was significantly slower in the MP28-treated group than in the control group (Figure 5A). No significant loss of body weight was observed in the MP28-treated group compared with the control group during the treatment period (Figure 5B). Thus, MP28 significantly inhibited tumor growth in this A549 xenograft model without causing overt toxicity.

## 3. Discussion

Antimicrobial/anticancer peptides (AMPs/ACPs) intended for pharmacological evaluation can be obtained from diverse natural or engineered origins [23,24]. Among ACP candidates derived from *Bryopsis plumosa*, we previously reported MP06 as a lead suitable for efficacy and mechanistic studies in cancer cell lines. MP28 was predicted to be an ACP using in silico tools and exhibited in vitro an anticancer efficacy comparable to that of MP06 [19]. Notably, MP28 exhibits hallmarks of membrane-active AMPs, with its cationic residues (Lys, Arg) poised for strong electrostatic interactions with the negatively charged, phosphatidylserine-enriched outer leaflet of cancer cell membranes [10,25]. MP28 contains an α-helical amphipathic domain (residues 3–16) flanked by flexible random-coil segments at both the N- and C-termini. This arrangement facilitates membrane insertion and enhances peptide stability under physiological conditions [26]. Proline residues and a balanced hydrophobic–cationic distribution further increase membrane affinity and improve stability in biological environments [27]. Moreover, MP28 comprises 27 amino acids and includes four cysteines capable of forming intramolecular disulfide bonds, yielding a loop-stabilized conformation that increases proteolytic resistance and may prolong bioactivity in vivo [28]. The relatively high tryptophan and arginine content supports deep bilayer penetration and mitochondrial membrane perturbation, consistent with previous findings that Trp/Arg-rich cationic peptides traverse lipid bilayers and trigger intrinsic apoptotic signaling [29,30]. Collectively, MP28 physicochemical and structural attributes suggest its potential as a selective, low-toxicity anticancer peptide.

Cytotoxicity analyses demonstrated that MP28 induced dose-dependent cell death in multiple cancer cell lines, while exhibiting minimal effects on normal human lung fibroblasts (MRC-5) and red blood cells. The IC_50_ for cancer cells (~10 μM) was substantially lower than the concentrations that cause hemolysis or cytotoxicity in normal cells, consistent with the concept of differential membrane selectivity between malignant and normal cells arising from lipid composition and membrane potential differences. Importantly, MP28 caused no or minimal hemolysis at 20 μM (the concentration sufficient for robust growth inhibition) and hemolysis remained ≤10% at all tested concentrations. 

Tumor metastasis is a key cancer progression driver, responsible for over 90% of cancer-related deaths, and poses a significant challenge for the clinical management of most patients with advanced disease [31]. In general, the motility of cancer cells is influenced by EMT, a program of cellular changes vital for embryonic development, wound healing, and the malignant progression of cancer [32]. We assessed vimentin expression (as a marker of EMT), an intermediate filament protein associated with mesenchymal cytoskeletal organization and cell motility [33]. Specifically, MP28 markedly reduced vimentin expression and the migration and invasion ability of lung cancer cells. MP28 appears to act as a potential EMT inhibitor that disrupts vimentin expression, and ultimately inhibits cancer cell growth, invasion, and migration. Nonetheless, the mechanisms underlying MP28’s EMT-inhibitory effects remain unclear and warrant further investigation.

Primary membrane-binding effects and short-term exposure (3 h) activated the intrinsic apoptotic pathway via caspase-9, a canonical hallmark of apoptosis [34]. This finding corroborates previous findings of peptide-induced caspase-9 activation in leukemia, esophageal squamous cell carcinoma, and breast adenocarcinoma models [35,36]. The distinct patterns of activated (cleaved) caspase-9 observed among A549, H460, and H1299 cells likely reflect intrinsic differences in their apoptotic signaling background, particularly their p53 status [37]. A549 and H460 cells harbor wild-type p53, whereas H1299 cells are p53-null. Because p53 transcriptionally regulates several key components of the intrinsic apoptotic pathway, including BAX, Bcl-2 and PUMA, p53-proficient cells are more effectively primed to undergo mitochondrial outer membrane permeabilization, cytochrome c release, and subsequent Apaf-1–caspase-9 apoptosome formation in response to stress stimuli [38,39]. Consistent with Figure 4B, A549 and H460 cells exhibited a more rapid and robust induction of cleaved caspase-9 following MP28 treatment, while p53-deficient H1299 cells showed less efficient or delayed apoptotic activation, resulting in a weaker activation signal. Thus, differences in p53 status may provide a plausible mechanistic explanation for the cell-line-specific patterns of caspase-9 activation detected in our NSCLC cells. Therefore, MP28 activity extends beyond physical membrane disruption to include mitochondrial stress in caspase-9 signaling.

ACPs integrate structural rigidity, high selectivity, low toxicity, and multimodal anticancer mechanisms, making them next-generation peptide-based therapeutic candidates [40]. The present study demonstrates that MP28 inhibits EMT and induces apoptosis via caspase-9 activation. Accordingly, MP28 may represent a potential candidate to overcome antitumor drug limitations in aggressive cancers.

## 4. Materials and Methods

### 4.1. Peptide Identification and Synthesis

The cDNA sequence of *Bryopsis plumosa* was obtained by PacBio sequencing, a single-molecule long-read sequencing method, from DNALINK (Seoul, Republic of Korea). Total RNA and cDNA were prepared as described previously and used for cDNA sequencing. Peptides that consisted of more than 50 amino acids were removed, and small peptides were used to predict ACPs. The possible ACP was predicted using a custom database prepared from peptide sequence information from CancerPPD (https://webs.iiitd.edu.in/raghava/cancerppd/cancerppd2.php, accessed on 12 April 2022), the CRI database (http://cancerresearch.org/peptide-database, accessed on 12 April 2022), and AntiCP 2.0. Among the 22 candidate anticancer peptides, MP28, a 27-amino acid peptide with the sequence CCKKPWLRCWRTCLPSRWQRERFGRKC-NH_2_, was selected and synthesized by DANDI Cure Co. (Republic of Korea, Figure S1) via solid-phase synthesis [19]. The purity and molecular mass of the peptides were determined using high-performance liquid chromatography (Shimadzu HPLC LabSolution, Kyoto, Japan). A stock solution (10 mM) of the peptide was prepared by dissolving it in distilled water and stored at −20 °C until further use. The 3D model of MP28 was generated using PEP-FOLD3 (https://bioserv.rpbs.univ-paris-diderot.fr/services/PEP-FOLD3/, accessed on 24 April 2024) and PyMOL 3.0 software (http://pymol.org, accessed on 24 April 2024).

### 4.2. Cell Culture and Proliferation Assay

Human lung fibroblast MRC5 and cancer cells (A549, H460, and H1299) were obtained from Korean Cell Line Bank (Seoul, Republic of Korea) and maintained in Dulbecco’s modified Eagle medium or RPMI-1640 (Hyclone, Cytiva, Marlborough, MA, USA) supplemented with 10% fetal bovine serum (FBS; Sigma-Aldrich, St. Louis, MO, USA) and 1% penicillin/streptomycin (Hyclone) at 37°C in a humidified atmosphere with 5% CO_2_.

For the proliferation assay, cells (5 × 10^3^) were seeded into 96-well plates and treated with MP28 at different concentrations in the culture medium. After 24 h, cells were incubated in a fresh medium containing 10% CCK-8 solution (Dojindo, Tokyo, Japan) at 37°C for 3 h. Cell proliferation was determined by measuring absorbance at 450 nm using a SpectraMax i3x (Molecular Devices, San Jose, CA, USA). To quantify the anticancer activity of lung cancer cell types, the half maximal inhibitory concentration (IC50), which is the concentration of MP28 required for a 50% inhibition of cell growth, was also measured.

Lung cancer cells were seeded at a density of 10^3^ cells in a 35 mm dish and cultured with the peptide for 7 days, until macroscopic colonies formed. Following incubation, the cells were washed and stained with 0.1% crystal violet for 30 min, and the colonies were imaged and counted. Cell morphology was observed and captured at ×10 magnification using an inverted light microscope.

### 4.3. Hemolytic Activity Assay 

A hemolytic activity assay was conducted to evaluate the potential hemolytic effects of MP28. The concentrations tested for MP28 were 0 (control), 10, 20, and 40 μM. In this assay, 1% Triton X-100, which causes 100% hemolysis of horse blood erythrocytes, was used as a positive control to indicate complete cell lysis. DW solution served as a negative control, indicating no hemolytic activity. 

### 4.4. Migration/Invasion and Wound-Healing Assay

Cell migration and invasion were assessed using Transwell chambers with 8 μm pores (BD Biosciences). Cancer cells (2 × 10^4^) were seeded in serum-free medium in the upper chamber, while medium containing 10% FBS was placed in the lower chamber. Matrigel-coated upper chambers were used for invasion assays. Following incubation, the migratory and invasive cells were fixed, stained with crystal violet, and imaged under a light microscope.

For the wound-healing assays, cancer cells were grown to >90% confluence in 6-well plates, and scratches were created using a pipette tip. After washing, the cells were incubated in a medium with 10% FBS for 18 h and 24 h. Wound closure was monitored using an inverted phase-contrast microscope (CKX53, Olympus, Shinjuku, Tokyo, Japan), and migration was quantified by measuring the distance between wound edges at multiple sites.

### 4.5. Apoptotic Cell Analysis by Flow Cytometry

The treated cells (1 × 10^6^) were harvested, washed with PBS, and collected by centrifugation. Cells were stained with Annexin V and propidium iodide (PI) solution (556547; BD Biosciences, San Jose, CA, USA), followed by incubation at 37 °C for 15 min. To investigate the effects of caspase-9 activation, cells treated with 20 μM MP28 peptide were stained with FITC-LEHD-FMK for 1 h at 37 °C in a 5% CO2 incubator. As a negative control, z-VAD-FMK was added to each experimental condition according to the manufacturer’s instructions (ab65615; caspase 9 FITC staining kit). The cells were resuspended in 300 μL of a washing buffer and were analyzed by a flow cytometer (Accuri C6 Plus; BD Biosciences, San Jose, CA, USA).

### 4.6. Animal Experiments

Xenograft tumors were generated by subcutaneous injection of A549 lung cancer cells (1 × 10^6^ cells/100 µL) into the right flanks of BALB/c female nude mice (5 weeks of age, *n* = 20). Mice were randomly divided into two groups and intratumorally injected with 20 mg/kg MP28 six times, every other day. The control mice were injected with equal volumes of DW. The tumor size was measured using a caliper (calculated volume = shortest diameter^2^ × longest diameter^2^) at 2- or 3-day intervals. The methods were performed in accordance with the relevant guidelines and regulations and were approved by the Animal Care and Use Committee (IACUC) of the National Marine Biodiversity Institute of Korea (Approval No.: MAB-004; approval date: 2 December 2024).

### 4.7. Statistical Analysis

The results were expressed as the mean ± standard error of the mean. All experiments were conducted in triplicate. Comparisons were performed using a two-tailed paired Student’s *t*-test. Dose–response curves for determining inhibitory concentration (IC50) values were drawn by applying a graphical fitting method. GraphPad Prism 10.3.1 was used for statistical analysis, and a *p*-value < 0.05 was regarded as statistically significant (indicated with an asterisk * in the corresponding figures as follows: * *p* < 0.05, ** *p* < 0.01, *** *p* < 0.001).

## Data Availability

The data that support the findings of this study are available from the corresponding author upon reasonable request.

## Supplementary Materials

The following supporting information can be downloaded at https://www.mdpi.com/article/10.3390/md23120481/s1, Figure S1: Chromatographic profiles and mass spectra for MP28 peptide.

## Abbreviations

The following abbreviations are used in this manuscript:

ACP	anticancer peptide
AMP	antimicrobial peptide
EMT	epithelial–mesenchymal transition
FBS	fetal bovine serum
FITC	fluorescein isothiocyanate
IACUC	Institutional Animal Care and Use Committee
IC50	half maximal inhibitory concentration
NSCLC	non-small-cell lung cancer
OS	overall survival
PBS	phosphate-buffered saline
PI	propidium iodide
