# Supplementary figures and images for: Induction of Apoptotic Cell Death in Non-Small-Cell Lung Cancer Cells by MP28 Peptide Derived from Bryopsis plumosa
Source: Mar Drugs. 2025 Dec 17;23(12):481. doi: 10.3390/md23120481 (PMC12734986; doi:10.3390/md23120481)

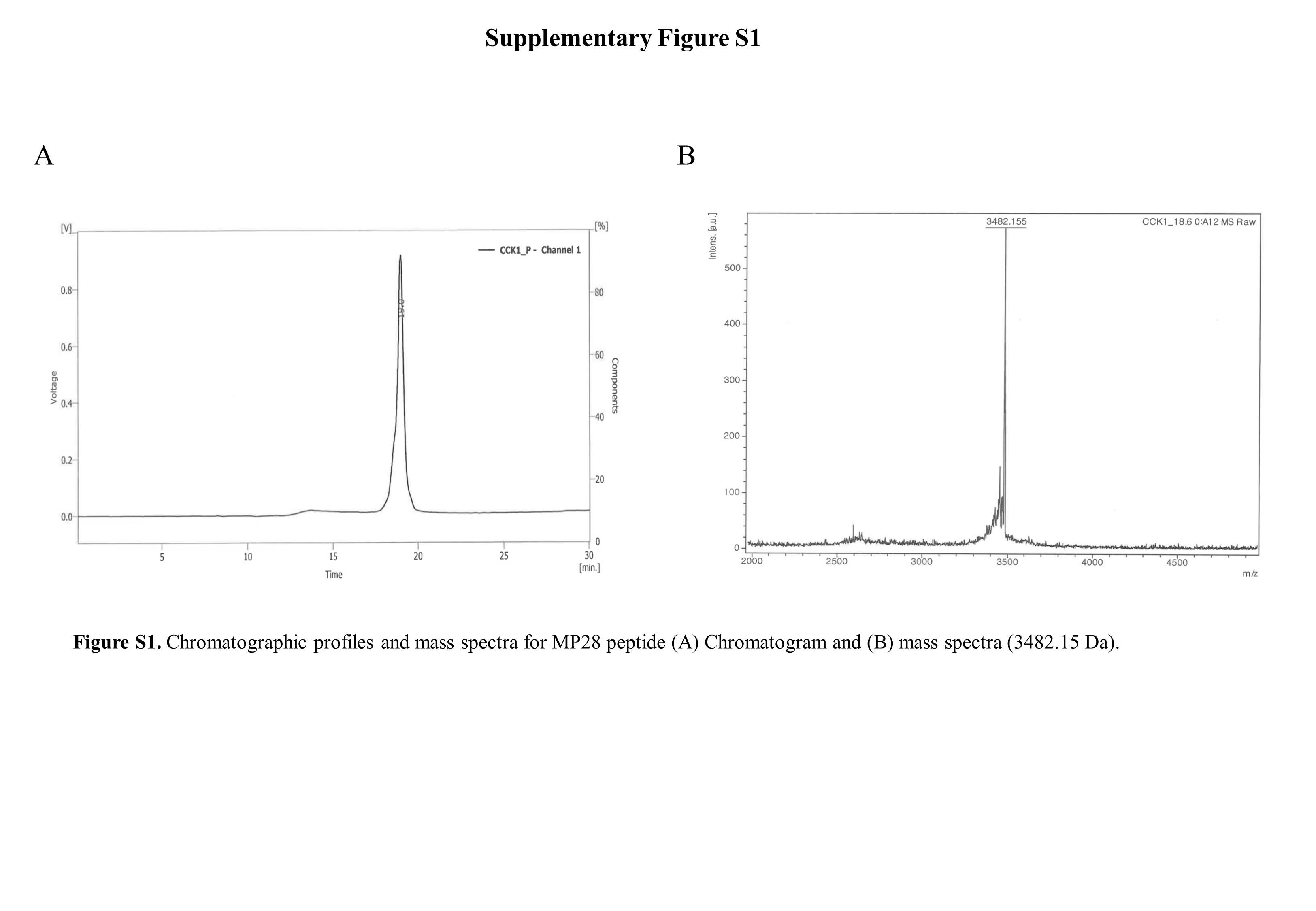

Supplement: Supplementary file 1 [file marinedrugs-23-00481-s001.zip › marinedrugs-4020070-supplementary.TIF]
